# Short Term Feeding of a High Fat Diet Exerts an Additive Effect on Hepatocellular Damage and Steatosis in Liver-Specific PTEN Knockout Mice

**DOI:** 10.1371/journal.pone.0096553

**Published:** 2014-05-12

**Authors:** Colin T. Shearn, Kelly E. Mercer, David J. Orlicky, Leah Hennings, Rebecca L. Smathers-McCullough, Bangyan L. Stiles, Martin J. J. Ronis, Dennis R. Petersen

**Affiliations:** 1 Department of Pharmaceutical Sciences, University of Colorado Denver Anchutz Medical Campus, Aurora, Colorado, United States of America; 2 Department of Pathology, University of Colorado Denver Anchutz Medical Campus, Aurora, Colorado, United States of America; 3 Arkansas Children's Nutrition Center, University of Arkansas for Medical Sciences, Little Rock, Arkansas, United States of America; 4 Department of Pediatrics, University of Arkansas for Medical Sciences, Little Rock, Arkansas, United States of America; 5 Department of Pathobiology, Cleveland Clinic, Cleveland, Ohio, United States of America; 6 Department of Pharmaceutical Sciences, University of Southern California, Los Angeles, California, United States of America; University of East Anglia, United Kingdom

## Abstract

**Background:**

Hepatospecific deletion of PTEN results in constitutive activation of Akt and increased lipogenesis. In mice, the addition of a high fat diet (HFD) downregulates lipogenesis. The aim of this study was to determine the effects of a HFD on hepatocellular damage induced by deletion of PTEN.

**Methods:**

12 Week old male flox/flox hepatospecific PTEN mice (PTEN^f/f^) or Alb-Cre controls were fed a HFD composed of 45% fat-derived calories (from corn oil) or a normal chow. Animals were then analyzed for hepatocellular damage, oxidative stress and expression of enzymes involved in fatty acid metabolism.

**Results:**

In the Alb-Cre animals, the addition of a HFD resulted in a significant increase in liver triglycerides and altered REDOX capacity as evidenced by increased GPX activity, decreased GST activity and decreased hepatic concentrations of GSSG. In addition, SCD2, ACLY and FASN were all downregulated by the addition of HFD. Furthermore, expression of PPARα and PPARα-dependent proteins Cyp4a and ACSL1 were upregulated. In the PTEN^f/f^ mice, HFD resulted in significant increased in ALT, serum triglycerides and decreased REDOX capacity. Although expression of fatty acid synthetic enzymes was elevated in the chow fed PTEN^f/f^ group, the addition of HFD resulted in SCD2, ACLY and FASN downregulation. Compared to the Alb-Cre HFD group, expression of PGC1α, PPARα and its downstream targets ACSL and Cyp4a were upregulated in PTEN^f/f^ mice.

**Conclusions:**

These data suggest that during conditions of constitutive Akt activation and increased steatosis, the addition of a HFD enhances hepatocellular damage due to increased CD36 expression and altered REDOX status. In addition, this work indicates HFD-induced hepatocellular damage occurs in part, independently of Akt signaling.

## Introduction

Non-alcoholic fatty liver disease (NAFLD) is a leading cause of liver disease in the United States today. A common phenotype of NAFLD is an environment characterized by pronounced hepatic lipid accumulation and enhanced oxidative stress. In a subset of NAFLD patients, symptoms progress to nonalcoholic steatohepatitis (NASH) and in this inflammatory environment a further subset will progress to fibrosis and ultimately cirrhosis [1], [2], [3], [4].

Many animal models of NAFLD utilize a long term feeding of a diet high in polyunsaturated fatty acids to induce hepatocellular steatosis. Frequently, additional hepatic insults such as cholesterol or oxidized low density lipoproteins are added as well [5], [6], [7]. In NASH, steatosis is frequently regarded as the first “hit” and is hypothesized to be the prerequisite for progression to steatohepatitis. A second, not yet definitively identified, “hit” is required for the progression to steatohepatitis. This second hit has been proposed to include cellular processes such as mitochondrial injury, oxidative stress, innate immunity or proinflammatory cytokines [8].

The phosphatase and tensin homolog deleted on chromosome 10 (PTEN)/Akt pathway is well documented in its ability to directly regulate *de novo* lipogenesis (DNL) in the liver [9]. PTEN is a dual specificity phosphatase possessing both lipid and protein phosphatase activity and is a member of the protein tyrosine phosphatase (PTP) family of phosphatases [10], [11]. PTEN negatively regulates Akt activation through its ability to dephosphorylate the 3-position phosphate from PtdIns (3,4,5) P_3_ to produce PtdIns (4,5) P_2_. Inactivation of PTEN leads to sustained Akt activation in both cellular and animal models. Hepatospecific deletion of PTEN (PTEN^f/f^) is an established model to examine the effects of a NASH-like condition [1]. In the liver, PTEN^f/f^ results in insulin hypersensitivity, hepatomegaly, triglycerides, and constitutive activation of DNL. As these mice age, a progression into steatohepatitis and ultimately hepatocellular carcinoma occurs in mice fed normal chow diets [12], [13]. PTEN expression in other organs and tissues is normal but there is an overall reduction in overall body fat [1], [3], [12], [13], [14].

In the present study, the effects of short term feeding of a HFD was used as a second hit and examined in a background of enhanced steatosis that occurs in PTEN^f/f^ mice. We demonstrate that addition of a HFD significantly exacerbates hepatocellular damage and oxidative stress in PTEN^f/f^ mice. Furthermore, HFD suppresses expression of *de novo* synthetic enzymes downstream of Akt and upstream of SREBP1. This study also provides additional insight into the mechanism of HFD-induced oxidative stress and delineates the relative contribution of the PTEN/Akt pathway in HFD-induced hepatocellular damage.

## Materials and Methods

### Animal model

PTEN^f/f^ mice and Alb-Cre mice on a C57Bl6/J background were bred as previously described [12]. Mice, 12 weeks of age in groups of six were fed a liquid HFD (45% fat derived calories from corn oil) (Bio-Serv, Frenchtown, NJ) or standard chow for six weeks. Upon completion of the study, animals were anesthetized via intraperitoneal injection with sodium pentobarbital and euthanized by exsanguination. Blood was collected from the inferior vena cava and plasma was separated through centrifugation @4°C and assayed for alanine aminotransferase (ALT) activity (Sekisui Diagnostics, P.E.I., Canada). Excised livers were weighed, sections of the liver caudate and median lobes collected, fixed in 10% neutral buffered formalin and embedded in paraffin for histological and immunohistochemical examination and prepared for hematoxylin and eosin staining. The remaining portion was subjected to differential centrifugation and subcellular fractionation as previously described [15]. All procedures involving animals were approved by the Institutional Animal Care and Use Committee of the University of Colorado and were performed in accordance with published National Institutes of Health guidelines.

### Western blotting

Proteins from either whole liver extracts or subcellular fractions were subjected to standard SDS-PAGE and transferred to PVDF (GE Healthcare, Picataway, NJ). Membranes were blocked for 60 minutes with a tris-buffered saline solution containing 1% Tween-20 (TBST) and 5% non-fat dry milk and probed overnight with primary antibodies directed according to Table S1. A horseradish peroxidase conjugated secondary (Jackson ImmunoResearch Inc. West Grove, PA) was then applied and membranes developed using ECL-Plus Reagent (GE Healthcare). Chemiluminescence was visualized using either film or a Storm 860 scanner from Molecular Dynamics (Sunnyvale, CA).

### Biochemical analysis

Liver triglycerides were measured in a 2∶1 chloroform:methanol extract of liver homogenate using a kit from Diagnostic Research Inc. Serum adiponectin was determined using serum diluted 1∶500 in assay buffer and an ELISA kit according to the manufacturer's protocol (Millipore, Billerica, MA). GSH, GSSG, GST activity, Gpx, TrxR activity and biotin hydrazide detection of carbonylated proteins were detected as previously described [16], [17], [18]. Protein concentrations were determined using a modified Lowry Protein Assay from Bio-Rad (Hercules, CA).

### Gene expression

Liver (50 mg) was taken from Cre and PTEN chow fed, and HFD-treated animals, and homogenized in 0.6 ml of RLT plus buffer (Qiagen) using a Precellys homogenizer (Bertin Technologies, Rockville, MD). Total RNA was isolated according to the manufacturer's instructions accompanying the RNeasy Plus RNA isolation kit (Qiagen). RNA samples were quantified by UV spectrometry, and total RNA was reversed transcribed using iScript cDNA synthesis (Bio-Rad Laboratories, Hercules, CA) as per manufacturer's instructions. Subsequent real-time PCR analysis was carried out using SYBR green and an ABI 7500 sequencing detection system (Applied Biosystems, Foster City, CA). Results were quantified using deltaC_T_ method relative to *GAPDH*. Gene specific primers for each gene tested are listed in Table S2.

### Statistical analysis

Relative densitometry of Western blots was quantified using ImageJ (http://rsb.info.nih.gov/ij/). Statistical analysis of data was performed using 1-way Analysis of Variance, 2-way Analysis of Variance or a student's t-test and Prism 4 for Windows (GraphPad Software, San Diego, CA). All data are expressed as mean +/− S.E. and *p* values <0.05 were considered significant.

## Results

### HFD results in an increase in hepatocellular damage and hepatic triglycerides in PTEN^f/f^ mice

To validate the penetrance of the Alb-Cre promoter, lysates were prepared from Chow and HFD fed Alb-Cre and PTEN^f/f^ livers and Western blotted for PTEN, pSer^473^Akt and total Akt. As shown in Figure S1A-C, PTEN^f/f^ results in a greater than 95% deletion of hepatic PTEN and a 3-fold increase in Akt phosphorylation.

The data presented in Table 1 describes the effect of hepatospecific PTEN deletion on HFD-induced hepatotoxicity. In the Alb-Cre animals, a HFD resulted in a mildly elevated serum ALT (1.15-fold). As expected, in the PTEN^f/f^ chow fed animals, serum ALT increased 6.7-fold when compared to Alb-Cre chow-fed animals. The addition of a HFD significantly elevated this marker of hepatocellular damage by 11.93, 10.3 and 1.81-fold when compared to chow-fed Alb-Cre, high fat fed Alb-Cre and chow-fed PTEN^f/f^ animals respectively. Overall, the addition of a HFD resulted in a significant increase in body weight in both genotypes. Comparing PTEN^f/f^ and Alb-Cre liver weights, PTEN^f/f^ resulted in significant increase in overall liver weight. Surprisingly, compared to chow-fed animals, HFD feeding resulted in decreased liver weight in both the Alb-Cre and the PTEN^f/f^ groups. This decrease in liver weight corresponded to decreased liver to body weight ratios in both HFD groups. In the chow-fed groups, PTEN^f/f^ resulted in a significant 6-fold increase in hepatic triglycerides compared to respective Alb-Cre controls. In both models, liver triglycerides were increased in the HFD-fed groups; (1.55-fold Alb-Cre, 1.66-fold PTEN^f/f-^).

**Table 1:** Biochemical and global analysis of serum and liver homogenates of chow-fed and HFD-fed Alb-Cre and PTEN^f/f^ mice.

		Alb-Cre			PTEN^f/f^			Two-Way ANOVA P value	
Parameter╪	Chow		HFD	Chow		HFD	Genotype	HFD	Interaction
ALT (U/L)	14.51±3.14^a^		16.70±3.23^b^	95.50±15.17^a,c^		173.05±25.65^b,c^	**<0.0001**	0.0516	0.0646
Liver weight (grams)	1.35±0.09^a^		0.95±0.05^a,b^	3.47±0.40^a^		2.60±0.21^b^	**<0.0001**	**0.0324**	0.4044
Change in body weight (grams)	2.97±0.42^a^		7.02±0.43^a,b^	2.34±0.35^c^		3.83±0.39^b,c^	**0.0002**	**<0.0001**	**0.0059**
Liver/Body Weight	4.57±0.08^a^		3.20±0.05^a,b^	12.26±0.89^a^		10.53±0.48^b^	**<0.0001**	**0.0438**	0.8728
Liver Triglycerides (µg/mg tissue)	0.002±0.0002^a^		0.0031±0.0007^b^	0.01±0.001^a,c^		0.02±0.001^b,c^	**<0.0001**	**0.0004**	**0.0035**

Serum ALT, liver weight, change in body weight, liver to body weight and liver triglycerides were determined as described in methods. Data are means± SEM as analyzed by two-way ANOVA with a Bonferroni *post hoc* analysis (Alb-Cre group compared to PTEN^f/f^ group). Means with a common superscript letter are significantly different (N = 6 mice/group (p<0.05)).

╪ Data are presented as mean± SEM. Statistical significance was determined by Two-Way ANOVA followed by Bonferroni post hoc analysis.

Letter with similar superscripts (a, b, c) denote significant difference of P<0.05.

### HFD results in an increase in hepatic steatosis but not fibrosis in PTEN^f/f^ mice

**Figure 1:**
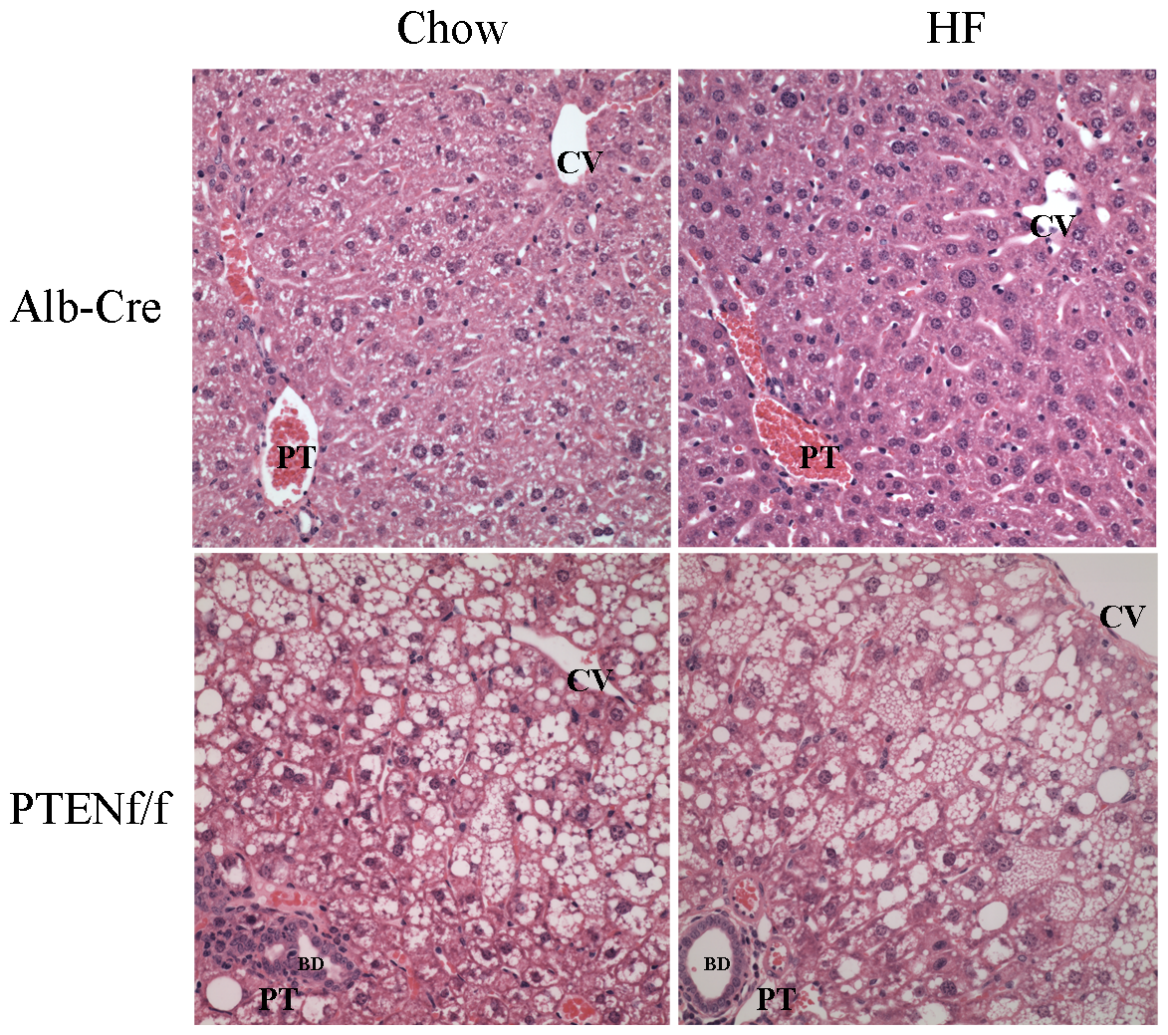
High fat diet results in increased lipid accumulation in PTEN^f/f^ mice. Hematoxylin and eosin staining of tissue sections from chow/HFD-fed Alb-Cre and PTEN^f/f^ mice. (CV, central vein, PT, portal triad, BD, bile ducts). Original Magnification 400X.

**Figure 2:**
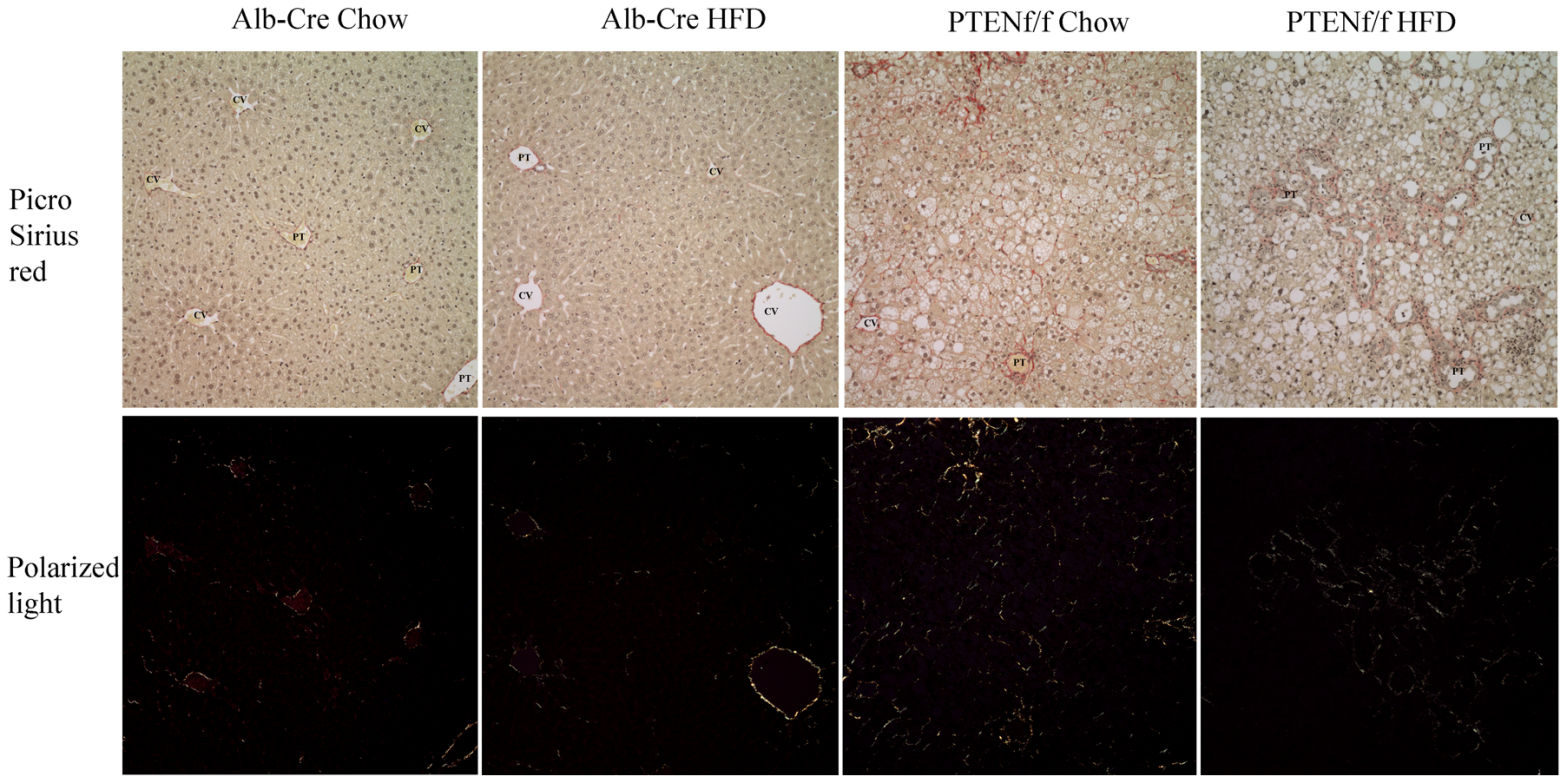
HFD does not alter fibrosis in PTEN^f/f^ mice. Picrosirius Red staining of tissue Hematoxylin and eosin staining of tissue sections from chow/HFD-fed Alb-Cre and PTEN^f/f^ mice. (A) White light. (B) Polarized light. (CV, central vein, PT, portal triad). Original Magnification 400X.

Given the finding that the addition of a HFD resulted in a significant increase in hepatocellular damage as determined by ALT, the effects of HFD in the PTEN^f/f^ background was examined in H&E stained liver sections. From Figure 1B, using hematoxylin and eosin staining, the addition of a HFD resulted in a mild increase in steatosis in Alb-Cre liver sections. In the chow-fed PTEN^f/f^ group, a dramatic increase in steatosis occurred primarily in hepatic zones 2 and 3. In addition, significant enlargement of the bile ducts (BD) was evident. The addition of a HFD resulted in a significant increase in overall hepatic steatosis in all three zones. Liver sections were then graded for steatosis and inflammatory foci [19]. In the Alb-Cre animals, no steatosis or inflammatory foci were evident in the chow-fed group. Addition of HFD resulted in a mildly increased in steatosis with no inflammatory foci (score 0–0.25). In the PTEN^f/f^ group, chow diet resulted in a significant increase in steatosis (score 2.25±0.38) with 0.775±0.35 lobular inflammatory foci/200X field. The HFD significantly increased both overall steatosis score (2.75±0.32) and inflammatory foci (1.3±0.24) in PTEN^f/f^ mice. As shown in Figure 2, no significant change in fibrosis was evident following HFD in either genotype but PTEN^f/f^ exhibited significantly more picrosirius red staining than the Alb-Cre animals.

### Effects of a HFD and hepatospecific PTEN deletion on oxidative stress and overall glutathione REDOX capacity

Reactive aldehydes are a validated measure of overall cellular oxidative stress in the liver [20]. In humans, lipid aldehydes are increased in NASH patients [21], [22], [23]. To determine the effects of a HFD on overall protein lipid peroxidation, whole cell extracts were treated with biotin hydrazide and examined by Western blotting. As shown in Table 2, HFD feeding did not result in an increase in overall protein carbonylation in the Alb-Cre groups. Compared to Alb-Cre controls, hepatospecific deletion of PTEN resulted in a significant decrease in protein carbonylation in the chow fed group. The addition of a HFD had very little effect in the Alb-Cre group but significantly increased overall protein carbonylation in PTEN^f/f^ animals. In hepatocytes, reactive aldehydes are removed via conjugation to glutathione [24]. Therefore, using whole hepatic tissue, the relative amounts of GSH, GSSG and ratio of GSH:GSSG was determined in all groups. From Table 2, in this system, HFD consumption did not significantly affect GSH in the Alb-Cre group. Compared to Alb-Cre controls, PTEN^f/f^ resulted in a significant 1.56-fold increase in GSH. Addition of HFD resulted in a decrease in GSH in PTEN^f/f^ group. Oxidation of glutathione occurs under conditions of oxidative stress. Comparing both models, deletion of PTEN resulted in a significant increase in GSSG. The addition of a HFD significantly reduced GSSG in the Alb-Cre group whereas GSSG was significantly increased in the PTEN^f/f^ group. A phenotype of increased cellular oxidative stress reflects significant decrease in the ratio of GSH:GSSG signifying decreased antioxidant capacity [25], [26]. Surprisingly, following HFD, GSH:GSSG was increased by 35% in the Alb-Cre mice suggesting a decrease in overall cellular oxidative stress. In the PTEN^f/f^ mice, HFD feeding resulted in a marked 40% reduction in GSH:GSSG indicating an increase in cellular oxidative stress. Compared to both groups of Alb-Cre mice, cellular redox ratios significantly decreased in the PTEN^f/f^ mice. 2-way ANOVA revealed an interaction between HFD and PTEN^f/f^. Combined, these data indicate that hepatospecific deletion of PTEN results in increased hepatocellular oxidative stress that is further exacerbated by the addition of HFD.

**Table 2:** Oxidative stress measurements in hepatic tissue isolated from chow/HFD-fed Alb-Cre and PTEN^f/f^ mice.

		Alb-Cre			PTEN^f/f^			Two-Way ANOVA P value	
Parameter╪	Chow		HFD	Chow		HFD	Genotype	HFD	Interaction
GSH (µmol/g tissue)	3.39±0.26^a^		3.37±0.24	5.29±0.44^a^		4.01±0.64	**0.0142**	0.1797	0.1923
GSSG (µmol/g tissue)	0.27±0.01^a^		0.17±0.01^a,b^	0.52±0.02^a^		0.61±0.03^b^	**<0.0001**	0.1633	**0.0011**
GSH:GSSG	12.64±0.76^a^		19.43±1.30^a,b^	10.23±0.76^c^		6.51±0.68^b,c^	**<0.0001**	0.1675	**0.0002**
GST activity (U/mg protein)	19.93±2.28^a^		13.89±1.09^a^	19.52±1.29^c^		14.59±0.94^c^	0.9181	**0.0007**	0.6944
GPX activity (U/mg protein)	17.98±0.83^a^		26.67±1.30^a,b^	15.46±1.25		16.85±0.75^b^	**<0.0001**	**0.0005**	**0.005**
TrxR activity (Percent Alb-Cre Chow)	100±12.23		108±4.23^a^	134.00±11.18		152.46±7.742^a^	**0.0013**	0.175	0.6356
GSTμ expression	100±10.16^a^		104.62±4.77	169.640±21.15^a,b^		104.70±10.16^b^	**0.0277**	**0.0494**	**0.0279**
GSTπ expression	100±13.76		103.83±4.23^a^	65.44±7.98		49.43±4.92^a^	**0.001**	0.5	0.283
GSTA4 expression	100±12.57		101.07±7.21^a^	79.96±4.05		58.26±4.98^a^	**0.0042**	0.2303	0.1881
Carbonylation (Percent Chow Alb-Cre)	100.00±7.28^a^		103.90±7.70^b^	53.59±4.86^a,c^		85.16±8.33^b,c^	**0.0007**	**0.0291**	0.0774

Data are means± SEM as analyzed by two-way ANOVA with a Bonferroni *post hoc* analysis (Alb-Cre group compared to PTEN^f/f^ group). Means with a common superscript letter are significantly different (N = 6 mice/group (p<0.05)).

╪ Data are presented as mean± SEM. Statistical significance was determined by Two-Way ANOVA followed by Bonferroni post hoc analysis. Letter with similar superscripts (a, b, c) denote significant difference of P<0.05.

### Analysis of the effects of PTEN^f/f^ and HFD on GST, GPx and TrxR activity

The data presented in Table 2 suggests that hepatospecific deletion of PTEN results in an increase in oxidative stress and altered glutathione metabolism. In human patients with livers staged as NASH, GST activity was increased [27]. In other studies however, GST activity was reduced during the progression of NAFLD to NASH [28]. To further elucidate these processes in our models, overall GST and GPx activity was assessed. Comparing each genotype, chow feeding did not significantly affect GST activity. In both groups, the addition of a HFD resulted in a significant decrease in GST activity. Examining GPx activity, compared to Alb-Cre chow-fed mice, PTEN^f/f^ significantly suppressed GPx activity. The addition of a HFD resulted in a significant 1.5-fold increase in GPx activity in the Alb-Cre group. Surprisingly, PTEN^f/f^ prevented HFD-induced increases in GPx activity compared to Alb-Cre control mice. An alternative indicator of oxidative stress is protein carbonylation [29]. Examining overall levels of carbonylation, PTEN^f/f^ significantly decreased protein carbonylation compared to chow fed Alb-Cre animals. Although the addition of HFD did not have a significant effect in Alb-Cre animals, in the PTEN^f/f^ group, carbonylation was increased 1.6-fold. The thioredoxin/thioredoxin reductase system assists in the antioxidant response by reducing oxidized cysteine residues [25]. PTEN has also been demonstrated to directly interact with thioredoxin 1 altering activity [30], [31]. Therefore, the effect of PTEN^f/f^ and a HFD on thioredoxin reductase activity was examined. From Table 2, a HFD had no effect in the Alb-Cre animals. In PTEN^f/f^, thioredoxin reductase activity was significantly increased when compared to respective Alb-Cre Chow fed as well as HFD fed animals.

**Figure 3:**
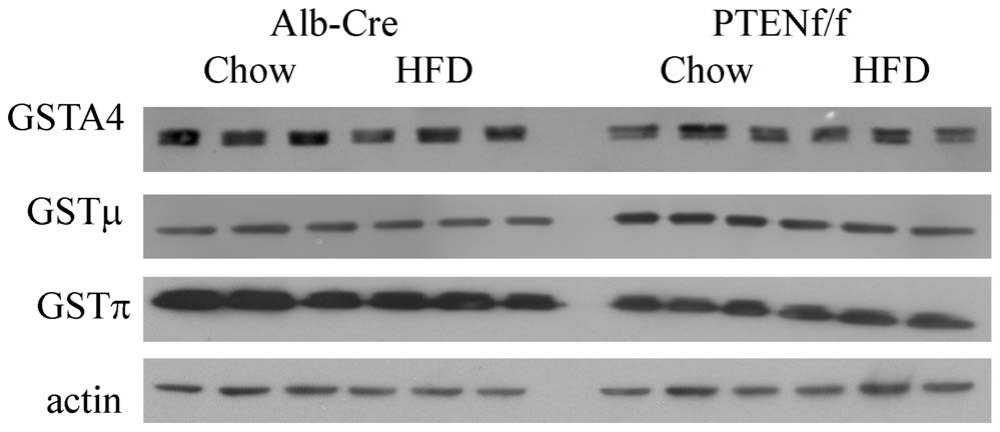
Effects of HFD on expression of GST's in Alb-Cre and PTEN^f/f^. Western blotting analysis of GSTA4, μ and μ using whole cell extracts isolated from chow/HFD-fed Alb-Cre and PTEN^f/f^ mice.

Based on our activity data, we sought to determine the specific isoforms of GST that are contributing to the observed changes in activity. From the Western blot and quantifications in Figure 3 and Table 2, a HFD did not significantly affect expression of GSTμ, π or A4 in the Alb-Cre mice. Comparing genotypes, PTEN^f/f^ resulted in a significant increase in overall GSTμ expression but surprisingly, expression of GSTπ and A4 was significantly decreased. Furthermore, the addition of a HFD resulted in only GSTμ expression. Using 2-way ANOVA, a significant interaction was observed for only GSTμ. All three isoforms tested exhibited genotype specific effects although not in the same direction. Overall, the data presented in Table 2 and Figure 3 PTEN^f/f^ induces cellular oxidative stress and may exert a selective effect on glutathione metabolism.

### Analysis of the effects of HFD and PTEN^f/f^ on serum adiponectin

As shown in Figure 1 and Table 1, the addition of a HFD resulted in a significant increase in both liver triglycerides and steatosis in the PTEN^f/f^ model. In NASH, serum adiponectin inversely correlate to body fat mass and negatively correlates with disease progression [32]. The relative levels of serum adiponectin have not been evaluated in PTEN^f/f^ mice with a HFD. As shown in Table 3, as expected given that PTEN^f/f^ mice have previously been reported to possess reduced visceral fat mass, in the chow-fed group, PTEN^f/f^ resulted in a 70% decrease in serum adiponectin [12]. Surprisingly, the addition of a HFD had no effect in the Alb-Cre groups but significantly increased serum adiponectin 2-fold in the PTEN^f/f^ group. This increase however, did not restore serum adiponectin to Alb-Cre chow fed levels. This suggests that although not directly measured, the increase in adiponectin in HFD PTEN^f/f^ correlates to an increase in body fat mass that would correlate with the decreased liver to body weight ratios found in HFD PTEN^f/f^ mice.

**Table 3:** Statistic analysis of the Western blots presented in Figure 2.

		Alb-Cre			PTEN^f/f^			P value	
Parameters (actin normalized)╪	Chow		HFD	Chow		HFD	vs genotype	vs HFD	Interaction
β-oxidation/fatty acid transport									
pAMPK	100±9.60		107.80±4.13	104.77±1.48		110.32±3.05	0.5638	0.2731	0.1276
total AMPK	100±13.64		109.04±1.79	116.39±5.21		105.97±3.86	0.4073	0.9309	0.237
pACC	100±9.30^a^		89.33±2.43^b^	252.19±12.07^a^		206.83±16.87^b^	**<0.0001**	**0.0399**	0.1677
total ACC	100±8.64^a^		108.90±9.84^b^	199.21±3.18^a^		178.21±4.76^b^	**<0.0001**	0.4218	0.0698
CPT1α	100±5.95^a^		92.67±5.69	76.0±1.84^a,b^		135.06±18.01^b^	0.3824	**0.0316**	0.103
PPARα	100±30.00^a^		128.98±18.34^b^	192.81±11.85^a^		174.10±38.65^b^	**0.033**	0.8524	0.3994
Cyp4a	100±0.72^a^		142.29±9.63^a,b^	123.23±11.41^a,c^		187.86±6.67^b,c^	**0.003**	**0.0002**	0.208
ACOX1	100±6.32		86.56±11.56	102.83±4.34^a^		83.76±1.60^a^	0.9984	**0.0483**	0.697
LFABP	100±4.74		107.88±0.72	106.94±6.28		80.71±4.02	0.5295	**0.0489**	0.2966
ACSL	100±8.76^a^		144.85±5.33^a^	168.98±17.84^a^		147.84±2.32	**0.0084**	0.2852	**0.0129**
serum adiponectin (µg/ml)	27.80±2.64^a^		28.79±1.77^c^	8.65±1.4^a,b^		15.63±1.73^b,c^	**<0.0001**	**0.0477**	0.1682
lipogenesis									
FASN	100±9.61^a^		82.67±4.26^b^	148.84±12.41^a^		137.01±7.13^b^	**0.0004**	0.1392	0.7647
ACLY	100±16.41^a^		28.32±10.06^a,b^	192.26±6.85^a^		159.32±15.82^b^	**<0.0001**	**0.0037**	0.1722
SCD-2	100±11.26^a^		67.64±2.3^a,b^	179.40±17.25^a,c^		97.25±10.92^b,c^	**0.0017**	**0.0012**	0.0676
SCD-1	100±7.85^a^		58.06±7.29^a,b^	161.40±6.39^a^		145.72±9.76^b^	**<0.0001**	**0.0066**	0.1359
PPARγ	100±2.20^a^		81.18±8.13^b^	150.19±8.59^a,c^		113.60±7.23^b,c^	**0.0002**	**0.0042**	0.2409
PGC1α	100±2.85		96.70±19.16	121.99±27.40		145.79±3.62	0.0683	0.5604	0.4452
nuclear transcription factors									
PPARγ	100±27.84^a^		228.28±67.94^b^	2363.87±419.19^a^		2889.97±321.36^b^	<0.0001	0.2547	0.477
PPARα	100±34.34^a^		147.82±43.40^b^	501.93±79.21^a^		830.64±108.96^b^	<0.0001	0.0323	0.0899
SREBP 1	100±3.16^a^		81.34±4.68^b^	118.17±5.22^a^		86.07±2.30^b^	0.0213	0.0002	0.1325
SREBP 2	100±5.77^a^		130.91±7.97^a^	57.55±18.94		108.68±12.90	**0.032**	**0.011**	0.4409
Nrf-2	100±10.61		75.5±3.37	71.2±7.28		76.4±8.62	0.1168	0.2563	0.0981

Data are means± SEM as analyzed by two-way ANOVA with a Bonferroni *post hoc* analysis (Alb-Cre group compared to PTEN^f/f^ group). All proteins were normalized to either actin (whole cell extracts) or lamin B1 (nuclear extracts). Means with a common superscript letter are significantly different (N = 3 mice/group (p<0.05)).

╪ Data are presented as mean± SEM. Statistical significance was determined by Two-Way ANOVA followed by Bonferroni post hoc analysis.

Letter with similar superscripts (a, b, c) denote significant difference of P<0.05.

### Analysis of effects of HF-diet and PTEN^f/f^ on expression of key enzymes involved in hepatic fatty acid metabolism

**Figure 4:**
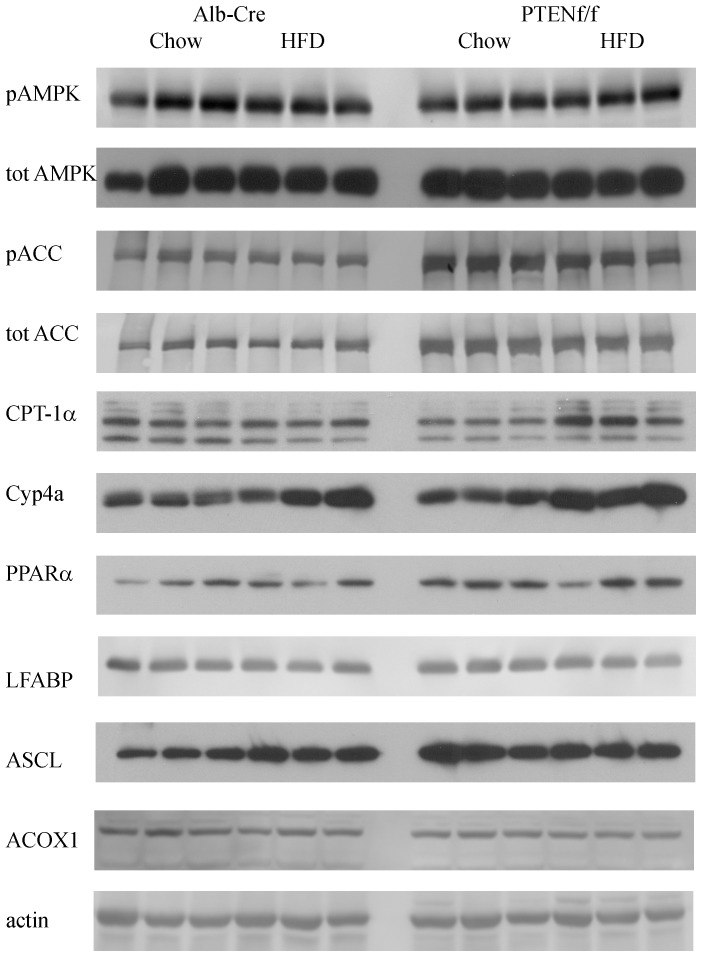
Effects of HFD on regulation of β-oxidation in Alb-Cre and PTEN^f/f^. Western blotting analysis of key enzymes involved in β-oxidation using whole cell extracts isolated from chow/HFD-fed Alb-Cre and PTEN^f/f^ mice.

In the liver, adiponectin binds the adiponectin receptors resulting in an increase in 5′ AMP activated phosphorylation (AMPK) on Thr^172^ [33]. Activated pThr^172^AMPK subsequently phosphorylates acetyl CoA Carboxylase (ACC) inhibiting ACC activity and decreasing malonyl CoA, an inhibitor of β-oxidation [34], [35]. AMPK signaling has not been examined under PTEN^f/f^ conditions. Whole cell extracts were prepared from hepatic tissue isolated from chow-fed/HFD-fed Alb-Cre and PTEN^f/f^ mice and probed for total and phosphorylated forms of AMPKα and ACC. From Figure 4, neither the addition of a HFD nor deletion of PTEN had an effect on AMPKα expression or phosphorylation indicating that there was not a compensatory β-oxidative response to increased lipid accumulation. Surprisingly, pSer^79^ACC was significantly increased corresponding to an increase in overall ACC expression in PTEN^f/f^ mice [13], [36]. Combined these data suggest that PTEN^f/f^ does not induce changes in AMPK but significantly effects its immediate downstream target ACC.

In murine models of diet induced obesity, PPARα expression has been show to increase [37]. Elevated pSer^79^ACC in both PTEN groups led us to further investigate by examining expression of PPARα and expression of the PPARα-dependent enzymes carnitine palmitoyltransferase (CPT1α), acyl CoA oxidase 1 (ACOX1), long chain Acyl CoA synthetase (ACSL1), cytochrome P450-4a (Cyp4a) and liver fatty acid binding protein (LFABP). From Figure 4, PPARα expression was mildly elevated by a HFD in the Alb-Cre group. Hepatospecific deletion of PTEN resulted in a significant increase in PPARα expression. Comparing chow and HFD feeding, expression of CPT-1α was not significantly altered in Alb-Cre animals. In the PTEN^f/f^ group, compared to chow-fed Alb-Cre animals, expression CPT1α was decreased. The addition of HFD resulted in increased CPT1α expression in PTEN^f/f^ animals. Examining expression of other PPARα-dependent proteins, changes in ACSL1 expression were similar to PPARα whereas Cyp4A was increased by high fat in both models. Surprisingly, both ACOX1 and LFABP were suppressed by HFD and did not exhibit an increase in expression in PTEN^f/f^ mice. Combined, these data indicate a selective effect concerning HFD and PTEN^f/f^ on cellular β-oxidative processes.

**Figure 5:**
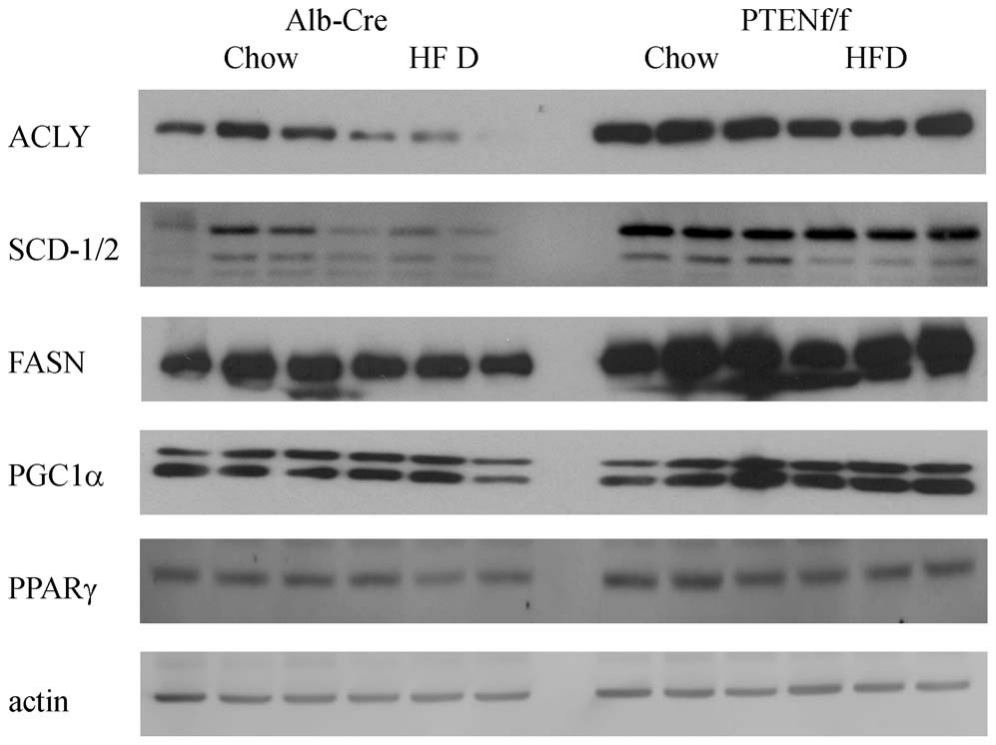
Effects of HFD on regulation of hepatic lipogenesis in Alb-Cre and PTEN^f/f^. Western blotting analysis of *de novo* lipogenic enzymes using whole cell extracts isolated from chow/HFD-fed Alb-Cre and PTEN^f/f^ mice.

It has previously been reported that hepatospecific deletion of PTEN results in increased expression of fatty acid synthetic enzymes [12], [14], [36]. The effects of a HFD on expression of fatty acid synthetic enzymes in PTEN^f/f^ mice have not been examined. From Figure 5 and Table 3, stearoyl CoA desaturase (SCD-1/2), fatty acid synthase (FASN), and ATP citrate lyase (ACLY) were all significantly decreased following HFD in the Alb-Cre groups. As expected, SCD-1, SCD-2, FASN and ACLY were significantly increased in the PTEN^f/f^ group. With the exception of SCD-1, the addition of a HFD resulted in a significant decrease in expression. This expression however, was elevated when compared to Alb-Cre HFD animals. Compared to chow-fed PTEN^f/f^ animals, HFD did not significantly decrease SCD-1 expression. PPARγ is directly regulated downstream of PTEN and regulates a variety of cellular processes including lipid metabolism, glucose metabolism, inflammatory responses and angiogenesis [13]. The addition of HFD resulted in a significant decrease in PPARγ expression in both Alb-Cre and PTEN^f/f^ mice. Comparing genotypes, PTEN^f/f^ resulted in a significant increase in PPARγ compared to Alb-Cre animals. PPARγ Coactivator 1α (PGC1α) also promotes *de novo* lipogenesis. The addition of HFD had no effect on PGC1α expression in Alb-Cre or PTEN^f/f^ mice. Comparing the two genotypes, PGC1α was elevated in PTEN^f/f^ animals.

**Figure 6:**
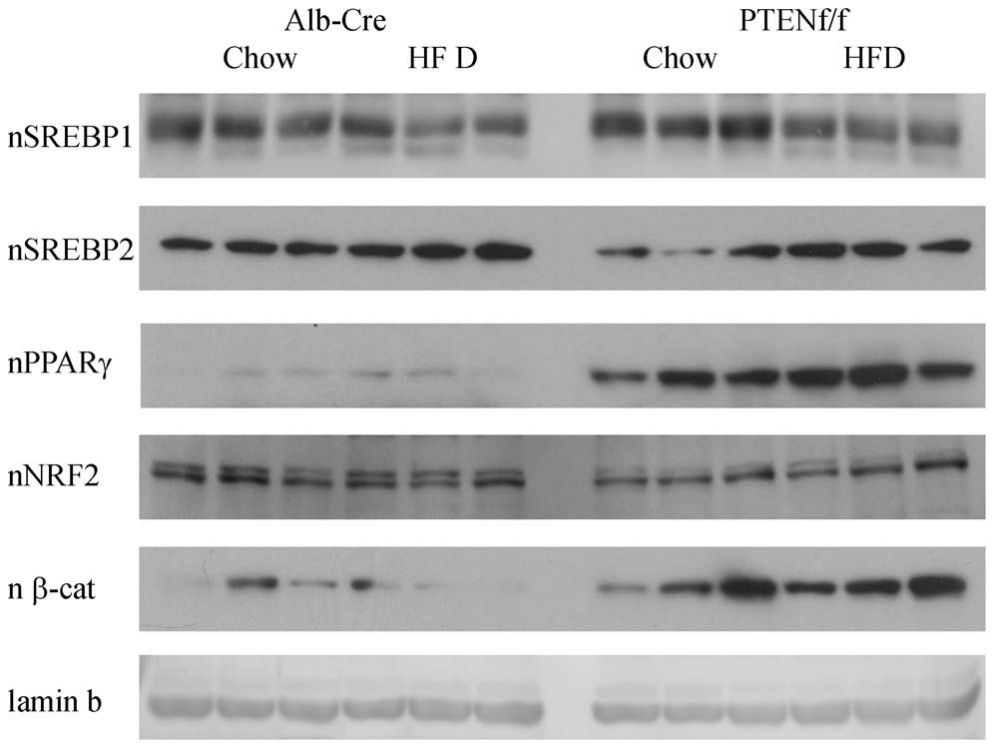
Effects of HFD on nuclear localization of metabolic transcription factors in Alb-Cre and PTEN^f/f^ mice. Western blotting analysis of metabolic transcription factors using nuclear fractions isolated from chow/HFD-fed Alb-Cre and PTEN^f/f^ mice.

Although substantial changes in expression of both β-oxidative as well as *de novo* lipogenic proteins is shown in Figures 4 and 5 the effects of HFD in combination with PTEN^f/f^ on nuclear localization of relevant transcription factors has not been described. Using Western blotting of nuclear extracts, nuclear localization of PPARγ, SREBP1, SREBP2, Nrf2 and β-catenin was evaluated (Figure 6, Table 3). Nuclear translocation of SREBP1 is a critical factor in the regulation of fatty acid synthetic proteins [38]. From Figure 6 and Table 3, nuclear localization of SREBP1 is decreased by HFD feeding in both models. PTEN is a direct regulator of PPARγ. As expected, PPARγ expression is dramatically upregulated by PTEN^f/f^ as well as HFD. From Table 2, there is evidence of increased oxidative stress in our model. Following nuclear localization, Nrf2 stimulates expression of a variety of oxidative stress related proteins [28], [39]. Following HFD, nNrf2 is decreased in Alb-Cre animals. Hepatospecific PTEN deletion resulted in a decrease in nNrf2 but HFD had no significant effect. In cells, nuclear localization of β-catenin is an indicator of increased cellular proliferation. Examining the Western blot, nuclear β-catenin is significantly increased in the PTEN animals irrespective of diet compared to Cre controls.

### Effects of HFD and PTEN deletion on expression of selected hepatic genes

As shown in Table 1, feeding a HFD resulted in a significant increase in hepatocellular damage in PTEN^f/f^. To further characterize the effects of PTEN^f/f^ and HFD, mRNA analysis of TNFα, CD14, IL-6 and IL-10 was performed using mRNA isolated from fresh frozen tissue sections. As shown in Table 4, in the Alb-Cre groups, although TNFα and CD14 trended upward, HFD did not result in a significant increase in either proinflammatory (TNFα, CD14, IL-6) or anti-inflammatory cytokines (IL-10). Compared to the Alb-Cre groups, PTEN^f/f^ resulted in a significant increase in both TNFα and CD14 but there was no significant change in either rIL-6 or IL-10. This suggests that in the PTEN model inflammation is increased independently of consumption of 6 weeks of HFD.

**Table 4:** mRNA expression analysis of selected cytokines and fatty acid metabolic proteins from tissue isolated from chow/HFD-fed Alb-Cre PTEN^f/f^ mice.

Table 4 mRNA analysis of selected genes		Alb-Cre			PTEN^f/f^			P value	
inflammation	Chow		HFD	Chow		HFD	vs genotype	vs HFD	Interaction
TNFα	0.02±0.14^a^		0.11±0.16^c^	0.41±0.14^a^		0.48±0.12^c^	**0.023**	0.605	0.974
CD14	0.09±0.09^a^		0.17±0.11^b^	0.41±0.09^a^		0.36±0.08^b^	**0.018**	0.862	0.521
IL-6	0.07±0.06		0.08±0.05	0.12±0.05		0.05±0.05	0.964	0.616	0.541
IL-10	0.02±0.01		0.03±0.01	0.03±0.01		0.01±0.01	0.794	0.955	0.118
lipid transport and metabolism									
PPARα	0.02±0.02^a,b^		0.07±0.02^b^	0.04±0.02^a,c^		0.08±0.12^c^	0.419	**0.034**	0.664
PPARγ	0.51±0.64^a^		0.76±0.74^b^	5.48±0.58^a^		6.41±0.58^b^	**<0.001**	0.374	0.609
CD36	0.01±0.02^a^		0.02±0.02^b^	0.11±0.01^a,c^		0.18±0.01^b,c^	**<0.001**	0.055	0.241
FATP2	0.30±0.11^a^		1.1±0.13^a,c^	0.40±0.11^b^		0.60±0.11^b,c^	0.188	**<0.001**	**0.027**

Data are means± SEM as analyzed by two-way ANOVA with a Holm-Sidak *post hoc* analysis (Alb-Cre group compared to PTEN^f/f^ group).

Values are mean (SEM); statistical analysis performed by two-way ANOVA followed by Holm-Sidak analysis.

Letter with similar superscripts (a, b, c) denote significant difference of P<0.05.

From Table 3, Western blotting analysis indicated a significant increase in both PPARα and PPARγ following PTEN deletion, to validate these data, mRNA analysis was performed. As shown in Table 4, mRNA expression of both PPARα and PPARγ are significantly increased in PTEN^f/f^ mice. This increase was further enhanced by the addition of a HFD. The lipid transporter CD36 is a direct target of PPARγ and is upregulated in mice fed diets rich in fatty acids [19]. Fatty acid transport protein 2 (FATP2) assists in hepatic free fatty acid uptake [40]. In our study, Western blotting for CD36 was not successful, therefore mRNA expression of lipid transporters CD36 and FATP2 was performed. From Table 4, PTEN^f/f^ resulted in a significant increase in CD36 but not FATP2. The addition of a HFD significantly increased FATP2 in both models but CD36 only in the PTEN^f/f^ model. Analysis of CD36 and FATP2 by two-way ANOVA indicated a significant interaction between HFD and PTEN^f/f^ with respect to FATP2 but not CD36. In summary, a HFD exerts differential effects on lipid transport proteins when combined with hepatospecific PTEN deletion.

## Discussion

The accumulation of fat is the first step in the progression of NASH. In this study, we utilized an initial hit of steatosis due to hepatospecific deletion of PTEN and followed it by the addition of a second hit in the form of a HFD over a short time course. Not surprisingly, in our Alb-Cre animals, the addition short term HFD only induced a mild accumulation of hepatic triglycerides and only demonstrated a trend in increased hepatocellular damage. This is an expected result, a longer duration of feeding is necessary to produce hepatocellular damage in normal mice [41], . In PTEN^f/f^ mice, a HFD significantly increased triglycerides, body weight and ALT. Comparing both genotypes, PTEN^f/f^ resulted in a dramatic increase in overall liver weight, liver:body weight ratios, hepatocellular damage (as evidenced by ALT) and hepatic triglycerides. As expected, HFD resulted in a significant increase in overall body weight. In this study, hepatic triglycerides, body weight, and ALT were significantly increased in the HFD PTEN^f/f^ group. This indicates that the effects of a HFD on these parameters are independent of the PTEN pathway and that there is a combinatorial effect in the mutant group.

Not surprisingly, adiponectin levels were decreased in the chow fed PTEN^f/f^ group. Previous measurement of fat mass in chow fed PTEN^f/f^ animals indicated a significant decrease in body fat [12]. What is surprising is the increase in serum adiponectin in the HFD PTEN^f/f^ group. A recent study using Lepr^db/db^ mice has identified adiponectin as an independent predictor of NASH [44]. With HFD feeding, increased hepatic steatosis, triglycerides as well as adiponectin (albeit to levels still well below the Alb-Cre mice) occurred in the PTEN^f/f^ model. Yet the increase in adiponectin did not correlate with phosphorylation of AMPK and expression of PGC1α in the PTEN^f/f^ groups. Furthermore, both phosphorylation and total expression of ACC levels was significantly elevated regardless of diet and AMPK phosphorylation. The mechanism of this is not known at this time.

In the Alb-Cre model, HFD promoted alterations in cellular REDOX homeostasis as evidenced by decreased GST activity, increased GPx activity and decreased GSSG concentrations. This is in agreement with other studies where HFD was fed for longer periods of time [45]. When we examined individual isoforms of GST (μ, π and A4) we did not detect significant differences in expression following HFD in the Alb-Cre groups. These proteins did exhibit genotype specific effects in the PTEN^f/f^ groups. Overall, expression of the GST isoforms tested did not correlate well with our activity data suggesting that other GST isoforms may be in part responsible for the observed differences in GST activity. When combined with a preexisting propensity for lipid accumulation (PTEN^f/f^), the overall REDOX capacity and GPx activity were suppressed yet GST activity exhibited the same effect regardless of genotype. Concurrently, TrxR activity and the concentrations of both reduced and oxidized glutathione significantly increased in the PTEN^f/f^ group. With the exception of GST activity, this is in agreement with a previous study that demonstrated increased levels of oxidative stress in the PTEN^f/f^ animals [46], [47]. Given the further suppression of overall REDOX capacity in the HFD PTEN^f/f^ model, it is not surprising that there was a significant increase in hepatic levels of carbonylation [48], [49]. What is surprising is that comparing the 2 genotypes, carbonylation was significantly suppressed in both PTEN^f/f^ groups despite increased oxidative stress. Furthermore, deletion of PTEN results in increased mitochondrial respiration [48]. Based on these data, we propose that increased mitochondrial respiration and mitochondrial oxidative stress results in increased resistance to carbonylation. The mechanism of resistance however remains to be elucidated.

Although we demonstrate an interaction between PTEN/HFD with respect to triglycerides and body weight, this interaction is not reflected in parameters concerning fatty acid synthesis. In our Alb-Cre model, expression of key fatty acid synthetic enzymes was suppressed by a HFD. In the PTEN^f/f^ model, rates of fatty acid synthesis and expression of fatty acid synthetic enzymes are significantly upregulated [12], [13]. This upregulation has been proposed to be directly downstream of Akt2 [9], [50]. Yet in these animals, albeit a modest effect, HFD still suppresses expression of FASN and ACLY indicating that HFD may alter FASN/ACLY expression downstream of Akt or that suppression of these enzymes by HFD is independent of Akt. Mammalian target of rapamycin 2 (mTORC2) phosphorylates Akt on Ser^473^ resulting in full Akt activation in the liver [51]. Using liver specific mTORC2 deletion, the addition of constitutively active Akt2 was not able to restore activation of hepatic lipogenesis [52]. Thus, in our study, downregulation of hepatic lipogenesis by a HFD may be due to alterations in mTORC2 signaling independent of Akt2 activation or downstream of Akt2.

In the PTEN^f/f^ mice, significant upregulation of SREBP1 has been demonstrated [14]. However, in our Alb-Cre and PTEN^f/f^ mice, HFD resulted in decreased nuclear accumulation of SREBP1. In the livers of SREBP1 overexpressing mice, SCD2 is upregulated [53]. Although both SCD isoforms are suppressed by the addition of a HFD in the Alb-Cre model, in the PTEN^f/f^ groups only SCD-2 is significantly suppressed following HFD feeding. SCD-2 is thought to have an important role in the synthesis of monounsaturated fatty acids during early skin and liver development [54]. Although trending downward, expression of SCD1 is not significantly changed suggesting possible alternative mechanisms of regulation that are either downstream of Akt, independent of SREBP1 or via alternative transcription factors in the PTEN^f/f^ mice.

The addition of a HFD induces PPARα and PPARα-dependent fatty acid β-oxidation [37]. In PPARα KO mice downstream genes such as ACSL and Cyp4a are significantly decreased following a HFD [37]. We find that expression of ACSL as well as Cyp4a correlate with an increase in PPARα expression in both the Alb-Cre HFD group as well as in both PTEN^f/f^ groups. Yet alternative PPARα targets such as ACOX1 and are only induced by the addition of HFD and not by PTEN^f/f^. Furthermore, LFABP is not induced in the Alb-Cre group following HFD and is suppressed in the PTEN^f/f^ animals. This suggests that PTEN^f/f^ exerts a selective effect on PPARα-dependent proteins.

In conclusion, this study clearly indicates that even short term feeding of a HFD and PTEN deletion have an additive effect on hepatocellular damage and steatosis. Thus, patients with NAFLD should avoid diets rich in PUFAs even in the short term. Although HFD decreases nuclear localization of SREBP1, this decrease is not sufficient to restore levels of most fatty acid synthetic enzymes to normal levels. Furthermore, our data clearly demonstrates that the effects of HFD on *de novo* lipogenesis occur downstream of Akt or via independent mechanisms such as changes in lipid transport. Given the additive effect of PTEN^f/f^ and HFD on hepatocellular damage, these data also provide support that both dietary lipids and lipids derived from *de novo* lipogenesis contribute to hepatocellular steatosis in PTEN^f/f^ mice. It also suggests that in PTEN^f/f^ mice, increased β-oxidative processes are not sufficient to reduce hepatic damage.

## Supporting Information

Figure S1 Effects of PTEN^f/f^ and HFD on PTEN signaling. (A) Western blotting analysis of PTEN, pSer473Akt and total Akt using whole cell extracts isolated from chow/HFD-fed Alb-Cre and PTEN^f/f^ mice. (B) Quantification of PTEN expression. (C) Quantification of Akt phosphorylation. Data are means± SEM as analyzed by two-way ANOVA with a Bonferroni *post hoc* analysis (Alb-Cre group compared to PTEN^f/f^ group). Means with a common superscript letter are significantly different (N = 3 mice/group (***p<0.001)).
(TIF)

Table S1 Antibodies used for Western blotting in this study.
(XLSX)

Table S2 Primers used for this study.
(XLSX)
