# Supplementary figures and images for: Short Term Feeding of a High Fat Diet Exerts an Additive Effect on Hepatocellular Damage and Steatosis in Liver-Specific PTEN Knockout Mice
Source: PLoS One. 2014 May 12;9(5):e96553. doi: 10.1371/journal.pone.0096553 (PMC4018288; doi:10.1371/journal.pone.0096553)

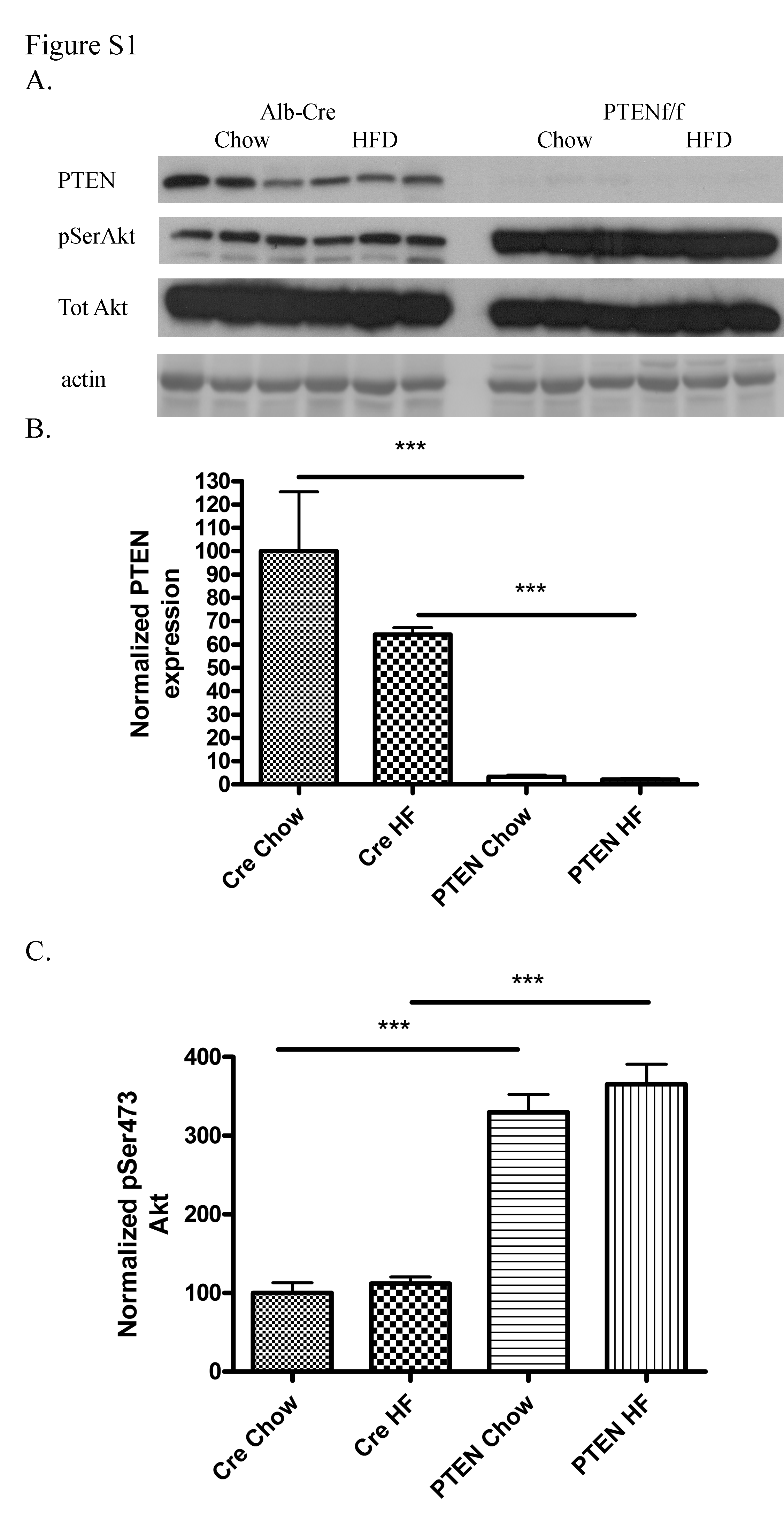

Supplement: Figure S1 — Effects of PTENf/f and HFD on PTEN signaling. (A) Western blotting analysis of PTEN, pSer473Akt and total Akt using whole cell extracts isolated from chow/HFD-fed Alb-Cre and PTENf/f mice. (B) Quantification of PTEN expression. (C) Quantification of Akt phosphorylation. Data are means± SEM as analyzed by two-way ANOVA with a Bonferroni post hoc analysis (Alb-Cre group compared to PTENf/f group). Means with a common superscript letter are significantly different (N = 3 mice/group (***p<0.001)). (TIF) [file pone.0096553.s001.tif]
